# Prevalence of pituitary dysfunction after aneurysmal subarachnoid hemorrhage: a systematic review and meta-analysis

**DOI:** 10.1186/s12883-023-03201-x

**Published:** 2023-04-20

**Authors:** Xiaowei Song, Shengnan Cong, Ming Zhang, Xiaokui Gan, Fan Meng, Baosheng Huang

**Affiliations:** 1 Department of Neurosurgery, Sir Run Run Hospital, Nanjing Medical University, Jiangsu, China; 2 Department of Nursing, Women’s Hospital of Nanjing Medical University (Nanjing Maternity and Child Health Care Hospital), Nanjing, China; 3 Department of Clinical Pharmacology, School of Pharmacy, Nanjing Medical University, Nanjing, China

**Keywords:** Pituitary hormone dysfunction, Aneurysmal subarachnoid hemorrhage, Global prevalence

## Abstract

**Background:**

Pituitary dysfunction (PD) is a common complication after aneurysmal subarachnoid hemorrhage (aSAH). The prevalence of PD varies widely at a global level and no recent meta-analysis is available. Therefore, the aim of our systematic review and meta-analysis was to summarize the updated estimates of worldwide prevalence of PD after aSAH.

**Methods:**

Scopus, Embase, Web of Science, and PubMed databases were used to comprehensively search the appropriate literature and a random-effects meta-analysis on the results of the available studies was performed. The heterogeneity in the prevalence estimates was evaluated by subgroup analysis in terms of types of PD, and acute and chronic phases of aSAH. The onset of PD within 6 months after aSAH was considered as acute, while that after 6 months was considered as chronic.

**Results:**

Twenty-seven studies with 1848 patients were included in this analysis. The pooled prevalence of PD in the acute phase was 49.6% (95% CI, 32.4-66.8%), and 30.4% (95% CI, 21.4-39.4%) in the chronic phase. Among the hormonal deficiencies, growth hormone dysfunction was the most prevalent in the acute phase, being 36.0% (95% CI, 21.0-51.0%), while hypoadrenalism was the most prevalent in the chronic phase, being 21.0% (95% CI, 12.0-29.0%). Among the six World Health Organization regions, the South-East Asia Region has the highest prevalence of PD in the acute phase (81.0%, 95%CI, 77.0-86.0%, *P* < 0.001), while the European Region had the highest prevalence of PD in the chronic phase (33.0%, 95%CI, 24.0-43.0%, *P* < 0.001). Moreover, single pituitary hormonal dysfunction occurred more frequently than the multiple one, regardless of acute or chronic phase.

**Conclusions:**

Almost half (49.6%) of the included patients with aSAH developed PD complication in the acute phase, while 30.4% of the patients developed them in the chronic phase. Although prevalence varies globally, the high healthcare burden, morbidity and mortality require greater awareness among clinicians.

**Supplementary Information:**

The online version contains supplementary material available at 10.1186/s12883-023-03201-x.

## Introduction

Aneurysmal subarachnoid hemorrhage (aSAH) is one of the most common forms of hemorrhagic stroke causing significant morbidity, such as neurological dysfunction and cognitive impairment, as well as mortality among patients [1]. The average annual incidence of aSAH in the global population is 6 to 10 per 100 000 people [2], and the average mortality rate is approximately 35% of patients in some countries [3]. Rapid diagnosis and medical intervention enable a survival rate of approximately 30%, and many patients can resume independent living [4]. The average age of onset of aSAH is 50 years and the highest incidence is between 40 and 60 years [5–9]. Because this age range represents a period of major responsibility for family and society and active creation of social value, the prognosis of patients in this age range is particularly important.

In recent years, an increasing number of aSAH survivors have been found to experience symptoms such as cognitive impairment, memory deterioration, fatigue, sexual dysfunction, and weight loss after treatment [5–15]. It was later confirmed that the origin of these symptoms was due to pituitary dysfunction (PD), which was then recognized as a common complication after aSAH [6, 10, 13, 16]. PD after aSAH seriously affects patients’ quality of life and social function and it may last for quite a long time [17]. Thus, more and more studies focused on the prevalence, early identification and prevention of PD after aSAH [18]. However, studies on the prevalence of PD after aSAH are based on only a few small cohorts, while studies on the acute and chronic phases of PD are scarce [19–23]. Therefore, a comprehensive statistical analysis of the prevalence of PD after aSAH is essential for the early diagnosis, early recognition of symptoms and early treatment of PD.

A systematic review and meta-analysis reported a pooled prevalence of PD of 49.3% in the acute phase after aSAH, being less, such as 25.6% in the chronic phase [24]. Another meta-analysis reported a PD prevalence after aSAH of 31% in the acute phase and 25% in the chronic phase [25]. These two studies have been published more than six years ago, and new related studies were published during this period. Additionally, these studies did not subdivide pituitary hormone dysfunction into adrenocorticotropic hormone dysfunction, gonadotropin dysfunction, and thyroid-stimulating hormone dysfunction. An effective guidance cannot be provided without the prevalence of each pituitary hormone dysfunction due to different treatments targeting different hormonal disorders. Thus, it is of utmost importance to update the results.

Hence, the aim of this systematic review is to summarize the updated estimate by analyzing the most recent literature on PD after aSAH. First, the prevalence of the acute and chronic phases of PD after aSAH was calculated, regardless of the diagnosis based on basal hormonal or stimulation experiments. Then, the prevalence of various types of PD in the acute and chronic stages was separately considered, including adrenocorticotropic hormone (ACTH) deficiency, growth hormone deficiency (GHD), thyroid-stimulating (TSH) hormone deficiency, gonadotropin (Gn) deficiency. Finally, the prevalence of PD in each WHO Regional Office and the prevalence of single/multiple pituitary hormone dysfunctions were analyzed.

## Methods

### Literature search

Medical subject heading (MeSH) terms combined with text words were used to maximize the search range of articles performed up to April 2023 using Scopus, Embase, Web of Science, and PubMed. Moreover, relevant studies were manually retrieved as a supplementation. The search text words used were the following: “hypopituitarism OR pituitary dysfunction OR impairment of pituitary function OR adenohypophysial dysfunction OR anterior pituitary deficiency OR corticotropin deficiency OR ACTH deficiency OR hypoadrenalism OR hypoadrenocorticism OR adrenocortical hypofunction OR GH deficiency OR GHD OR growth hormone deficiency OR TSH deficiency OR thyrotropic dysfunction OR thyrotropin deficiency OR hypothyroidism OR gonadotropin deficiency OR hypogonadism OR prolactin disturbance OR hypothalamic-pituitary-adrenal axis OR HPA OR corticotropic axis deficit OR somatotropic axis OR pituitary-thyroid axis OR PTA OR gonadotropic axis OR diabetes insipidus” AND “SAH OR subarachnoid hemorrhage”. All retrieved documents were imported into Endnote X9 (Thomas Reuters 2019) to facilitate the subsequent literature screening.

### Inclusion and exclusion criteria

Studies included in this meta-analysis met the following conditions: (1) articles specifying the criteria for the diagnosis of aSAH, which was confirmed by CT scan and digital subtraction angiography, or articles explaining the location of the aneurysms. (2) Articles that include the diagnostic criteria and the prevalence of at least one of the following diseases: PD, GHD, hyperprolactinemia and diabetes insipidus, as well as the deficiency of ACTH, TSH and Gn. (3) Patients without endocrine dysfunction before aSAH. (4) Patients who are＞18 years old. (5) Only English-language studies.

The exclusion criteria were the following: (1) reviews, letters, case reports, conference abstracts and commentaries or articles without the availability of the original text. (2) Duplicate publications of the included studies. (3) Articles in which the prevalence of the disease was not provided or could not be calculated.

### Study selection

Titles or abstracts of publications suspected of meeting the eligibility criteria for this systematic review were selected for a detailed analysis. Then, two authors carefully reviewed the full text and appendix. The inclusion was made by two authors after reaching the consensus. In cases of disagreement a third author was involved in the discussion, and the inclusion was allowed after agreement among the three authors.

### Quality assessment

The Joanna Briggs Institute Prevalence Critical Appraisal Tool [26] was used to assess the quality of the study in the articles that met the full-text inclusion criteria. This tool includes ten questions answered with Yes, No, Unclear, and Not/Applicable. All studies were assessed by two authors (A and B) independently and a third author (author C) was involved to resolve any disagreements.

### Data extraction

Two authors filled the data extraction form together. According to this form, the data of the included articles were manually extracted and cross-checked by the two authors (A and B) separately. The latest article of multiple articles describing the same case series containing consistent data was used. If not, the earliest published articles was used due to the presence of recall bias. The prevalence rate measured by the stimulation test was preferentially adopted [27], and if not, the prevalence rate measured by the basal hormone test was used [8]. The disagreements on the extracted data were resolved by discussion or by the involvement of a third author (author C).

### Study characteristics

The following information was collected: authors of the article, year of publication, country, sample size, gender ratio, age, study design, World Federation of Neurological Surgeons Scale grade, Glasgow Coma Scale score, Hunt-Hess grade, Fisher grade or modified Fisher grade, aneurysm location, aneurysm treatment, and duration of patients’ follow-up. Then the countries of the individual study populations were classified according to the World Health Organization regional office [28], which include Regional Office for Africa (AFRO), Pan American Health Organization (PAHO), Regional Office for South-East Asia (SEARO), Regional Office for Europe (EURO), Eastern Mediterranean Regional Office (EMRO), and Western Pacific Regional Office (WPRO). The location of the aneurysm was categorized as an anterior circulation aneurysm and a posterior circulation aneurysm.

### Outcome measures

The primary outcome in this study was the prevalence of PD in the acute and chronic phases. The acute phase corresponded to the occurrence of symptoms associated with PD within the first 6 months after aSAH, while the chronic phase corresponded to the occurrence of symptoms associated with PD after the first 6 months of aSAH onset [18]. The secondary outcomes were the deficiency of each of the following hormones: ACTH, GH, TSH, Gn, prolactin, cortisol, or testosterone.

The prevalence of both PD and each hormone deficiency after aSAH in each included article was calculated by dividing the number of patients with a certain hormonal deficiency by the total of patients receiving the corresponding hormone testing experiment at the same point. The actual number of follow-up patients was considered as the denominator when calculating the frequency of pituitary dysfunction and each hormone deficiency in the subsequent follow-up because of the loss or death of patients during the follow-up. However, the original number of cases at the time of the enrollment was used as the denominator for the calculation of the prevalence rate at follow-up if the number of follow-up cases was not reported, regardless of the loss of patients during the follow-up.

### Statistical analysis

All statistical and sensitivity analyses were performed using Stata (version 16.0; StataCorp). All studies were stratified by the acute and chronic phases of PD. Subsequently, two groups in each stratification were identified according to the cut-off points: 3 months and 1 year. The global pooled prevalence of PD with inverse‐variance weights obtained from a random‐effects meta‐analysis model was computed using the metaprop command in Stata, which showed the prevalence value and 95% CIs. The heterogeneity was assessed using the *I*^*2*^ statistic, which ranged from 0 to 100%. An *I*^*2*^ index of 25% or lower was defined as a low degree of heterogeneity, 26–50% as a moderate degree of heterogeneity, and greater than 50% as a high degree of heterogeneity. A random-effects meta-analysis was used to calculate the overall pooled prevalence of PD after aSAH throughout this study because of the high heterogeneity (expected and observed). Finally, the source of heterogeneity in two sets of primary outcomes, i.e., the prevalence of PD, was estimated by subgroup analysis in terms of WHO Regional Office and single/multiple pituitary hormone deficiencies. A value of *P* < 0.05 was considered statistically significant.

## Results

### Search results

**Fig. 1:**
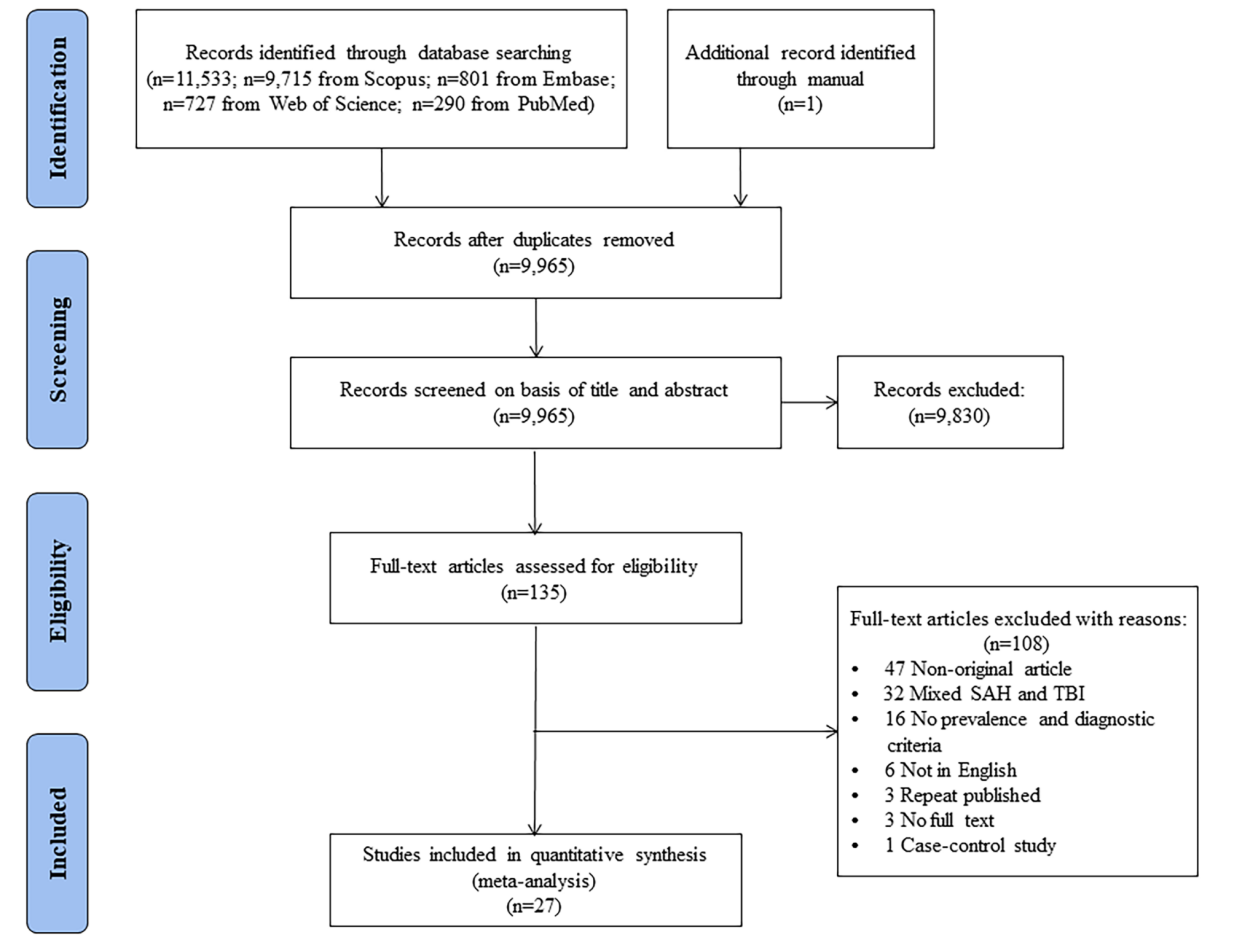
Flowchart of the search strategy

A total of 11,534 records (9715 in Scopus, 801 in Embase, 727 in Web of Science, 290 in PubMed and 1 from manually retrieved from the references of the articles collected using search engines) were identified through the initial systematic search, and among them, 1569 were removed because they were duplicates. Then, the abstracts and titles of the remaining 9965 articles were evaluated. A total of 135 articles were chosen according to our inclusion and exclusion criteria for the next stage consisting of the analysis of the full-text. The full-text analysis resulted in the exclusion of 108 studies for the reasons shown in Fig. 1. Finally, 27 studies were included in the final meta-analysis. The process of the systematic literature search is shown in the flow diagram in Fig. 1.

### Study characteristics

The included studies were published from 2004 to 2022, and the number of patients ranged from 20 to 417 per study, with a total of 1848 patients. According to the zoning of the WHO Regional Office, a total of 21 of these studies were performed in the EURO [8, 10, 13, 14, 19, 21–23, 29–41], 3 in the SEARO [42–44], 2 in the PAHO [45, 46], and 1 in the WPRO [47]. Among these studies, 22 had prospective study designs, and 5 were cross-sectional studies. A total of 740 patients had anterior circulation aneurysms, 146 of which aneurysms were in the posterior circulation, and 24 were mixed types, although some studies did not provide the location of the aneurysms. All relevant information for the included studies is listed in Table 1.

**Table 1:** Characteristics of the studies included in the analysis

Author (year)	Sample size, n (M/F)	Country	WHO Regional Office	Age (mean or median)	Design	WFNS (mean or median)	GCS (mean or median)	Hunt & Hess (mean or median)	Fisher (median)	Aneurysm location		Treatment	
										Anterior circulation	Posterior circulation	microsurgical clipping	endovascular occlusion
Aimaretti 2004 [29]	40 (14/26)	Italy	EURO	51.0 ± 2.1	prospective	NR	NR	NR	NR	NR	NR	NR	NR
Dimopoulou 2004 [10]	30 (14/16)	Greece	EURO	50 ± 13	prospective	NR	NR	2	2	24	6	10	20
Kreitschmann 2004 [13]	40 (14/26)	Germany	EURO	43.8 ± 7.6	cross sectional	NR	NR	2	3	26	8	NR	NR
Aimaretti 2005 [23]	32 (12/20)	Italy	EURO	51.9 ± 2.2	prospective	NR	NR	NR	2	NR	NR	NR	NR
Tanriverdi 2007 [19]	22 (11/11)	Turkey/Spain	EURO	47·9 ± 3·3	prospective	NR	NR	2	2	NR	NR	22	0
Klose 2010 [21]	62 (14/48)	Denmark	EURO	49	cross sectional	NR	NR	2	3	53	9	33	29
Jovanovic 2010 [38]	93 (30/63)	Serbia	EURO	48.0 ± 1.1	cross sectional	NR	NR	NR	NR	84	9	NR	NR
Lammert 2011 [39]	26 (6/20)	Germany	EURO	49.3	prospective	NR	NR	2	3	NR	NR	7	19
Schneider 2011 [8]	417 (139/278)	Germany	EURO	50.2 ± 11.6	cross sectional	NR	NR	2	NR	NR	NR	NR	NR
Lammert 2012 [40]	24 (4/20)	Germany	EURO	49.5 ± 14.5	prospective	NR	NR	2	3	NR	NR	6	18
Dutta 2012 [44]	60 (37/23)	India	SEARO	44.9 ± 13.1	prospective and retrospective	NR	NR	NR	NR	60	0	NR	NR
Lanterna 2013 [22]	26	Italy	EURO	53.5 ± 13.1	prospective	NR	NR	2	2	13	NR	18	8
Blijdorp 2013 [30]	43 (15/28)	Netherlands	EURO	56.6 ± 11.7	prospective	2	NR	NR	NR	24	19	6	37
Pereira 2013 [46]	66 (22/44)	Brazil	PAHO	48.3 ± 13.8	prospective	NR	13.8 ± 2.5	2	3	NR	NR	54	12
Karaca 2013 [33]	20 (12/8)	Turkey/Spain	EURO	47.6 ± 13	prospective	NR	NR	2	2	NR	NR	20	0
Khursheed 2013 [42]	73 (37/36)	India	SEARO	56 ± 13.5	prospective	3	NR	NR	3	NR	NR	NR	NR
Kronvall 2014 [14]	51 (8/43)	Sweden	EURO	55	prospective	NR	NR	2	3	NR	NR	13	38
Hannon 2015 [37]	100 (39/61)	Ireland	EURO	53	prospective	NR	NR	2	3	NR	NR	NR	NR
Tölli 2015 [41]	46(8/38)	Sweden	EURO	58.3 ± 10.5	prospective	NR	7.4 ± 3.9	4	4	36	11	NR	NR
Khajeh 2015 [36]	84 (28/56)	Netherlands	EURO	55.8 ± 11.9	prospective	2	13	NR	NR	49	35	17	66
Kronvall 2016 [35]	51 (8/43)	Sweden	EURO	55	prospective	NR	NR	NR	NR	NR	NR	13	38
Goto 2016 [47]	59 (19/40)	Japan	WPRO	58.0 ± 13.5	prospective	2	NR	3	NR	48	8 (both 3)	41	17 (both 1)
Vieira 2016 [45]	92 (33/59)	Brazil	PAHO	48.5	prospective	1	15	2	3	83	9	75	17
Tölli 2017 [32]	35 (8/27)	Sweden	EURO	57.4 ± 9.9	prospective	NR	7.9 ± 4.2	3	4	28	6 (both 3)	NR	NR
Giritharan 2017 [31]	100 (32/68)	UK	EURO	57 ± 10	cross sectional	1	NR	NR	4	72	10 (both 18)	67	19
Jaiswal 2017 [43]	100 (38/62)	India	SEARO	43.6	prospective	NR	NR	NR	NR	95	5	NR	NR
Robba 2022 [34]	56 (14/42)	Italy/ Russia	EURO	56.3 ± 11.0	prospective	2.0 ± 1.6	11.6 ± 4.0	2.0 ± 1.4	NR	45	11	16	40

NR: not reported

### Quality assessment

The quality of most studies was considered as moderate. Patients representing the aSAH population were recruited from Neurosurgical centers of large hospitals or Tertiary care centers. Most studies provided detailed inclusion and exclusion criteria, allowing the results of the present study to be representative of this population. Sample sizes were adequate in 3 studies (12%), but the rest of the studies failed to recruit enough patients because of the low prevalence of aSAH (6 to 10 per 100 000 people). Full quality assessment is shown in Table 2.

**Table 2:** Quality assessment of included studies

Author(years)	1.Was the sample representative of the target population?	2.Were study participants recruited in an appropriate way?	3.Was the sample size adequate?	4.Were the study subjects and the setting described in detail?	5.Was the data analysis conducted with sufficient coverage of the identified sample?	6.Were objective, standard criteria used for the measurement of the condition?	7.Was the condition measured reliably?	8.Was there appropriate statistical analysis?	9.Are all important confounding factors/subgroups/differences identified and accounted for?	10.Were subpopulations identified using objective criteria?
Aimaretti 2004	Yes	Unclear	No	Yes	Yes	Yes	Yes	Yes	No	Not applicable
Dimopoulou 2004	Yes	Yes	No	Yes	Yes	Yes	Yes	Yes	No	Yes
Kreitschmann-andermahr 2004	Yes	Yes	No	Yes	Yes	Yes	Yes	Yes	No	Not applicable
Aimaretti 2005	Yes	Unclear	No	No	Yes	Yes	Yes	Yes	No	Not applicable
Tanriverdi 2007	Yes	Unclear	No	No	Yes	Yes	Yes	Yes	No	Yes
Klose 2010	Yes	Yes	No	Yes	No	Yes	Yes	Yes	No	Yes
Jovanovic 2010	Yes	Yes	No	Yes	Yes	Yes	Yes	Yes	Yes	Yes
Schneider 2011	Yes	Yes	Yes	Yes	Yes	Yes	Yes	Yes	No	Not applicable
Lammert 2011	Yes	Yes	No	Yes	Yes	Yes	Yes	Yes	No	Not applicable
Lammert 2012	Yes	Yes	No	Yes	Yes	Yes	Yes	Yes	No	Not applicable
Dutta 2012	Yes	Yes	No	Yes	Yes	Yes	Yes	Yes	Yes	Yes
Pereira 2013	Yes	Yes	No	Yes	Yes	Yes	Yes	Yes	Yes	Yes
Blijdorp 2013	Yes	Yes	No	Yes	Yes	Yes	Yes	Yes	Yes	Yes
Karaca 2013	Yes	Unclear	No	No	Yes	Yes	Yes	Yes	No	Not applicable
Khursheed 2013	Yes	Yes	No	Yes	Yes	Yes	Yes	Yes	No	Not applicable
Lanterna 2013	Yes	Yes	Yes	Yes	Yes	Yes	Yes	Yes	Yes	Yes
Kronvall 2014	Yes	Yes	Yes	Yes	Yes	Yes	Yes	Yes	No	Not applicable
Tolli 2015	Yes	Yes	No	Yes	Yes	Yes	Yes	Yes	No	Not applicable
Hannon 2015	Yes	Yes	No	Yes	Yes	Yes	Yes	Yes	No	Not applicable
Khajeh 2015	Yes	Yes	No	Yes	Yes	Yes	Yes	Yes	Yes	Yes
Goto 2016	Yes	Yes	No	Yes	Yes	Yes	Yes	Yes	Yes	Not applicable
Kronvall 2016	Yes	Unclear	No	No	Yes	Yes	Yes	Yes	No	Not applicable
Vieira 2016	Yes	Yes	No	Yes	No	Yes	Yes	Yes	Yes	Yes
Giritharan 2017	Yes	Yes	No	Yes	Yes	Yes	Yes	Yes	Yes	Yes
Jaiswal 2017	Yes	Yes	No	Unclear	Unclear	Unclear	Unclear	Unclear	No	Not applicable
Tolli 2017	Yes	Yes	No	Yes	Yes	Yes	Yes	Yes	No	Yes
Robba 2022	Yes	Yes	No	Yes	Yes	Yes	Yes	Yes	No	Not applicable

### Outcome measures

#### Pooled prevalence of PD in the acute and chronic phases after aSAH

**Fig. 2:**
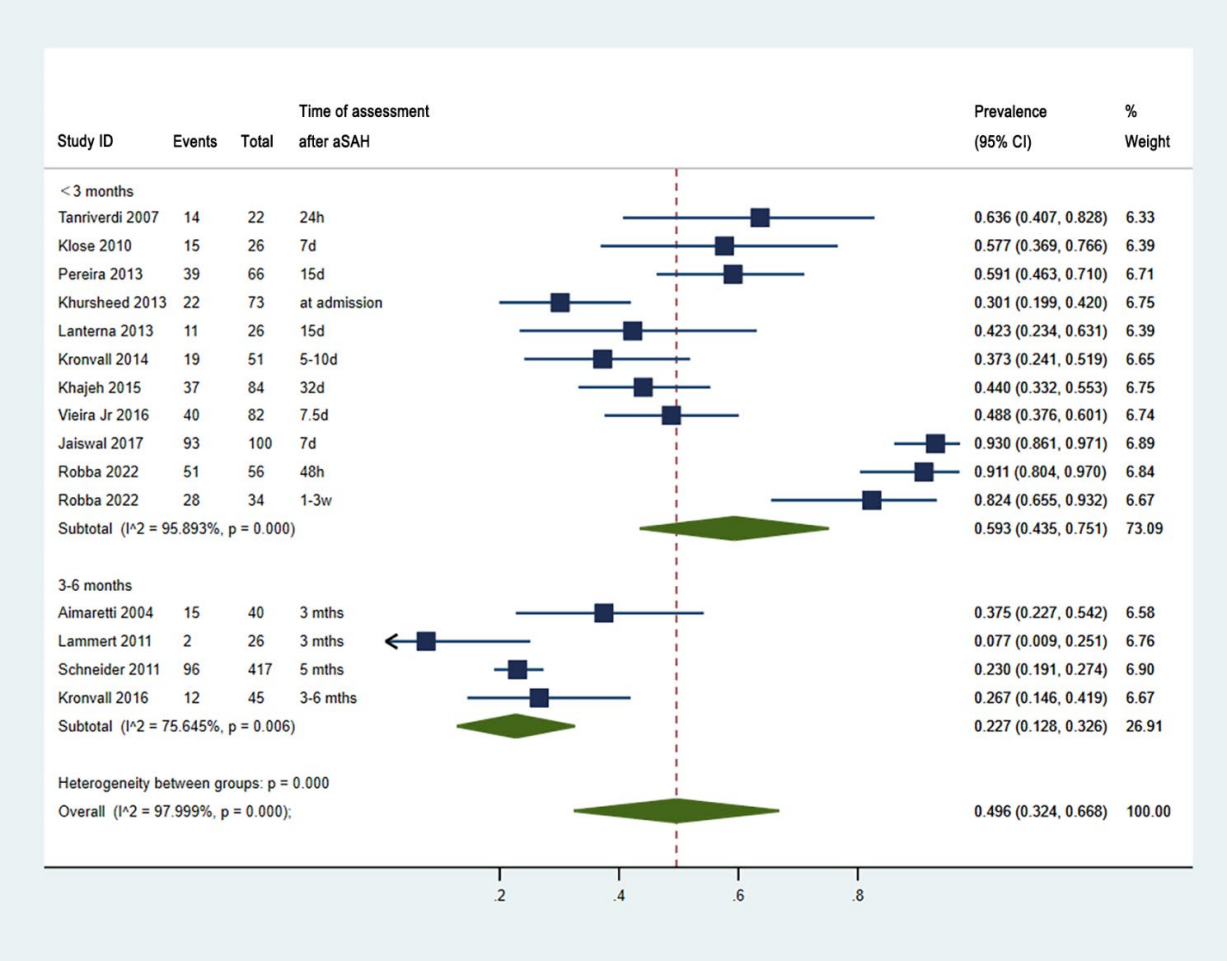
Pooled prevalence of PD in the acute phases after aSAH. CI, confidence interval

A total of 14 articles [8, 14, 19, 21, 22, 29, 34–36, 39, 42, 43, 45, 46] assessed the PD after aSAH in the acute phase (Fig. 2). The prevalence of PD after aSAH within 6 months in a total of 1148 patients was 0.50, with an estimated to range from 0.32 to 0.67 (*I*^*2*^ = 98.0%, *P* < 0.001). The subtotal prevalence of PD within 3 months was 0.59 (95% CI, 0.44–0.75, *I*^*2*^ = 95.9%, *P* < 0.001) and 0.23 between 3 and 6 months (95% CI, 0.13–0.33, *I*^*2*^ = 75.6%, *P* = 0.006).The 95% confidence interval of the prevalence within 3 months and 3–6 months had no overlap, as shown in Fig. 2; thus, the prevalence of PD within 3 months was significantly higher than that within 3–6 months (*P* < 0.001).

**Fig. 3:**
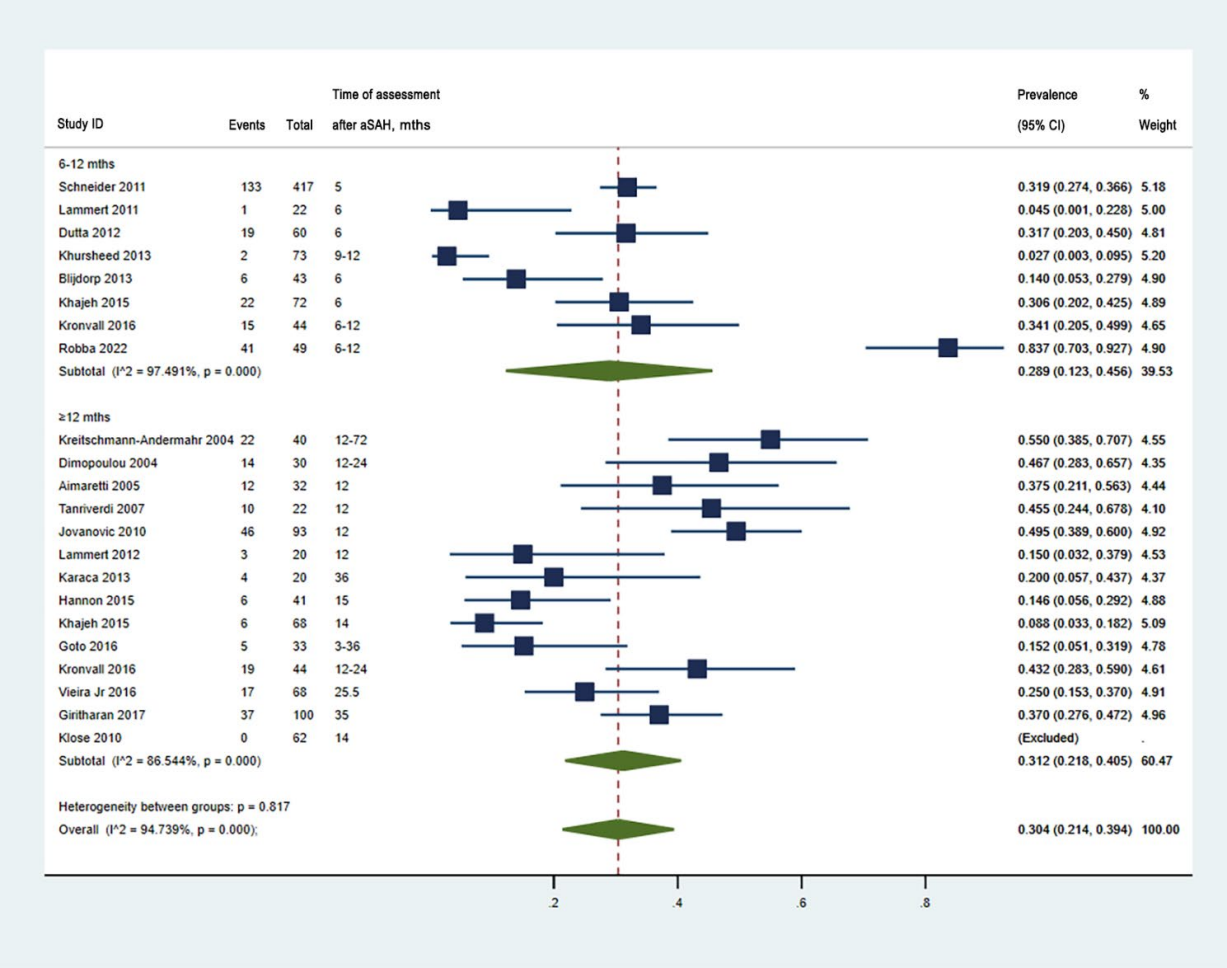
Pooled prevalence of PD in the chronic phases after aSAH. CI, confidence interval

Similarly, 20 articles [8, 10, 13, 19, 21, 23, 30, 31, 33–40, 42, 44, 45, 47] with a total of 1453 patients evaluated PD after aSAH in the chronic phase (Fig. 3). The prevalence of PD after aSAH after 6 months was 0.30, with an estimated range from 0.21 to 0.39 (*I*^*2*^ = 94.7%, *P* < 0.001). The subtotal prevalence of PD was 0.29 during 6–12 months (95% CI, 0.12–0.46, *I*^*2*^ = 97.5%, *P* < 0.001), while it was 0.31 when assessed after 12 months (95% CI, 0.22–0.41, *I*^*2*^ = 86.5%, *P* < 0.001). The prevalence rates of PD increased over time after 6 months but were not statistically significant (*P* = 0.817), indicating that the increase in the prevalence was not remarkable. The sensitivity analysis of the pooled prevalence of PD after aSAH is shown in Additional file 1 and Additional file 2.

### Global analysis of PD

The prevalence of each hormone deficiency in the acute and chronic phases was calculated by the comprehensive analysis of the included literature.

As regards the acute phase, a prevalence of ACTH deficiency of 0.15 (95% CI, 0.09–0.21, *I*^*2*^ = 90.0%, *P* < 0.001) was calculated in 15 studies [14, 19, 21, 22, 29, 32, 34–36, 39, 41, 43, 45, 46] that included 739 patients. The prevalence of GHD evaluated by 12 studies [14, 19, 21, 29, 34–36, 39, 43, 45, 46] with a total of 632 patients was 0.36 (95% CI, 0.21–0.51, *I*^*2*^ = 94.6%, *P* < 0.001). A total of 15 studies including 786 patients [14, 19, 21, 29, 32, 34–36, 39, 41–43, 45, 46] assessed a prevalence of TSH deficiency of 0.17 (95% CI, 0.09–0.24, *I*^*2*^ = 94.7%, *P* < 0.001). Thirteen studies [14, 19, 21, 29, 34–36, 39, 42, 43, 45, 46] with a total of 705 patients evaluated a prevalence of Gn deficiency of 0.33 (95% CI, 0.21–0.44, *I*^*2*^ = 93.4%, *P* < 0.001). The prevalence of hyperprolactinemia was 0.12 (95% CI, 0.07–0.16, *I*^*2*^ = 69.3%, *P* = 0.001) calculated in 12 studies [14, 19, 21, 29, 34, 35, 39, 42, 43, 45, 46] with a total of 621 patients. The 95% confidence intervals of the prevalence of GHD or Gn deficiency and the remaining ACTH deficiency or hyperprolactinemia had no overlap (Table 3); thus, the prevalence of GHD or Gn deficiency was significantly higher than that of ACTH deficiency and hyperprolactinemia.

**Table 3:** Global Analysis and Subgroup Analysis of Pituitary Dysfunction in the acute phases

Variable	No. of Articles	No. of Cases	No. of Participants	Prevalence (95% CI)	Heterogeneity		Subgroup difference
					Q test	*I*^*2*^, %	
**Global Analysis for classification of PD**							
ACTH deficiency	15	121	739	0.15 (0.09, 0.21)	*P*＜0.001	90.03%	NA NA NA NA NA
GH deficiency	12	215	632	0.36 (0.21, 0.51)	*P*＜0.001	94.57%	
TSH deficiency	15	124	786	0.17 (0.09, 0.24)	*P*＜0.001	94.66%	
Gn deficiency	13	238	705	0.33 (0.21, 0.44)	*P*＜0.001	93.37%	
Hyperprolactinemia	12	64	621	0.12 (0.07, 0.16)	*P* = 0.001	69.28%	
**Subgroup analysis of PD**							
WHO region							*P*＜0.001
ARFO	none	none	none	none	none	none	
PAHO	2	79	148	0.54 (0.46, 0.62)	NA	NA	
SEARO	2	115	173	0.81 (0.77, 0.86)	NA	NA	
EURO	11	301	827	0.47 (0.28, 0.65)	*P*＜0.001	97.07%	
EMRO	none	none	none	none	none	none	
WPRO	none	none	none	none	none	none	
Type							*P* = 0.674
Single	11	175	606	0.28 (0.20, 0.35)	*P*＜0.001	78.55%	
Multiple	11	175	606	0.25 (0.12, 0.37)	*P*＜0.001	95.04%	

CI: confidence interval, NA: not applicable

As regards the chronic phase, the prevalence of ACTH deficiency was 0.21 (95% CI, 0.12–0.29, *I*^*2*^ = 91.21%, *P* < 0.001) which was evaluated in 880 patients of 19 studies [10, 13, 19, 21, 23, 31–33, 35–40, 45, 47]. A total of 22 studies [10, 13, 19, 21, 23, 30–40, 44, 45, 47] that included 1018 patients evaluated a prevalence of GHD of 0.18 (95% CI, 0.14–0.22, *I*^*2*^ = 63.40%, *P* < 0.001). The prevalence of TSH deficiency calculated in 1062 patients of 22 studies [10, 13, 19, 21, 23, 31–40, 42, 44, 45, 47] was 0.05 (95% CI, 0.02–0.07, *I*^*2*^ = 41.94%, *P* = 0.070). The prevalence of Gn deficiency calculated in the same 1062 patients of the same 22 studies [10, 13, 19, 21, 23, 31–40, 42, 44, 45, 47] was 0.14 (95% CI, 0.09–0.19, *I*^*2*^ = 82.57%, *P* < 0.001). Hyperprolactinemia after 6 months was evaluated in 19 studies [13, 19, 21, 23, 31–35, 37–40, 42, 44, 45, 47] that included 918 patients, and the prevalence was 0.03 (95% CI, 0.01–0.04, *I*^*2*^ = 0.00%, *P* = 0.481). Among these hormonal deficiencies, the 95% confidence intervals of the prevalence of ACTH deficiency or GHD or Gn deficiency and the remaining TSH deficiency or hyperprolactinemia have no overlap suggesting that the prevalence of the deficiency of these three hormones was higher than that of TSH deficiency or hyperprolactinemia (Table 4). The amount of acticles on diabetes insipidus was too small; thus, no further analysis was performed after statistics.

**Table 4:** Global Analysis and Subgroup Analysis of Pituitary Dysfunction in the chronic phases

Variable	No. of Articles	No. of Cases	No. of Participants	Prevalence (95% CI)	Heterogeneity		Subgroup difference
					Q test	*I*^*2*^, %	
**Global Analysis for classification of PD**							
ACTH deficiency	19	124	880	0.21 (0.12, 0.29)	*P*＜0.001	91.21%	NA NA NA NA NA
GH deficiency	22	180	1018	0.18 (0.14, 0.22)	*P*＜0.001	63.40%	
TSH deficiency	22	33	1062	0.05 (0.02, 0.07)	*P* = 0.070	41.94%	
Gn deficiency	22	106	1062	0.14 (0.09, 0.19)	*P*＜0.001	82.57%	
Hyperprolactinemia	19	22	918	0.03 (0.01, 0.04)	*P* = 0.481	0.00%	
**Subgroup analysis of PD**							
WHO region							*P*＜0.001
ARFO	none	none	none	none	none	none	
PAHO	1	17	68	0.25 (0.15, 0.37)	NA	NA	
SEARO	2	21	133	0.05 (0.02, 0.09)	NA	NA	
EURO	18	396	1219	0.33 (0.24, 0.43)	*P*＜0.001	93.12%	
EMRO	none	none	none	none	none	none	
WPRO	1	5	33	0.15 (0.05, 0.32)	NA	NA	
Type							*P*＜0.001
Single	18	223	892	0.24 (0.16, 0.31)	*P*＜0.001	90.73%	
Multiple	18	55	892	0.07 (0.05, 0.10)	*P* = 0.062	43.19%	

CI: confidence interval, NA: not applicable

### Subgroup analysis for PD

The location of the WHO Regional Office where the included studies on PD after aSAH were included were EURO, SEARO, WPRO, and PAHO, and the related articles analyzed the prevalence of PD. Studies from the remaining two regions were not available.

Of the 15 studies, 11 studies [8, 14, 19, 21, 22, 29, 34–36, 39] with a total of 827 patients calculated a prevalence of PD of 0.47 (95% CI, 0.28–0.65, *I*^*2*^ = 97.07%, *P* < 0.001) in the EURO. As regards the remaining 4 studies, 2 studies [45, 46] in the PAHO with a total of 148 patients had a calculated prevalence of 0.54 (95% CI, 0.46–0.62), and the other 2 studies [42, 43] in the SEARO had a calculated prevalence of 0.81 (95% CI, 0.77–0.86) with a total of 173 patients. A statistically significant difference on the prevalence of PD in the acute phase was found among these three regions (Table 3, *P* < 0.001).

Of the 22 studies, 18 studies [8, 10, 13, 19, 21, 23, 30, 31, 33–40] with a total of 1219 patients calculated a prevalence of PD of 0.33 (95% CI, 0.24–0.43, *I*^*2*^ = 93.12%, *P* < 0.001) in the EURO. As regards the remaining 4 studies, 2 studies [42, 44] in the SEARO with a total of 133 patients had a calculated prevalence of 0.05 (95% CI, 0.02–0.09), and one study [45] from PAHO with a total of 68 patients had a calculated prevalence of 0.25 (95% CI, 0.15–0.37). Finally, one study [47] in the WPRO had a calculated prevalence of 0.15 (95% CI, 0.05–0.32) with a total of 33 patients. A statistically significant difference on the prevalence of PD in the chronic phase was found among these four regions (Table 4, *P* < 0.001).

As regards the acute phase, the random effects pooled meta-analysis performed on 11 studies [14, 19, 29, 34–36, 39, 43, 45, 46] with a total of 1212 patients showed an overall prevalence of single pituitary hormone dysfunction of 0.28 (95% CI, 0.20–0.35, *I*^*2*^ = 78.55%, *P* < 0.001) and an overall prevalence of multiple pituitary hormone dysfunctions of 0.25 (95% CI, 0.12–0.37, *I*^*2*^ = 95.04%, *P* < 0.001). Table 3 shows that the prevalence of single pituitary hormone dysfunction was higher than that of multiple pituitary hormone dysfunction, although not statistically significant.

As regards the chronic phase, the random effects pooled meta-analysis performed on 18 studies [10, 13, 23, 30, 31, 33–40, 42, 45, 47] with a total of 1784 patients showed an overall prevalence of single pituitary hormone dysfunction of 0.24 (95% CI, 0.16–0.31, *I*^*2*^ = 90.73%, *P* < 0.001) and an overall prevalence of multiple pituitary hormone dysfunction of 0.07 (95% CI, 0.05–0.10, *I*^*2*^ = 43.19%, *P* = 0.062). Table 4 shows that the 95% confidence interval of the prevalence of single and multiple pituitary hormone dysfunction had no overlap; thus, the prevalence of single pituitary hormone dysfunction was significantly higher than that of multiple pituitary hormone dysfunction.

## Discussion

Our meta-analysis demonstrated that the prevalence of PD after aSAH in the acute phases decreased over time and tended to be stable in the chronic phases, which was consistent with previous studies [6]. In details, the prevalence of PD within 3 months was relatively high, up to 59.3%, which was the first pooled prevalence found to the best of our knowledge. Can et al. [25] revealed that temporary and reversible endocrine changes in the early stages of aSAH (within 3 months) can interfere with the assessment of PD. This may have contributed to a higher prevalence of PD than it actually was. Then the overall prevalence in the acute phase affected by the prevalence of PD 3 months after aSAH was as high as 49.6%, which was similar to the results of Robba et al. [24], who reported a prevalence rate of 49.3% in PD after aSAH patients in the acute phase. The prevalence rate of 22.7% in PD between 3 and 6 months was comparable to that of PD in the chronic phase (overall 30.4%, 28.9% during 6–12 months and 31.2% after 12 months, respectively) although a slight increase over time was observed, but without statistical significance. These results were almost the same as those of the study of Can et al. [25] showing a prevalence of 31% and 25% in PD from 3 to 6 months and after 6 months of aSAH onset, respectively. Our speculation on the above findings was that most patients showing PD complication between 3 and 6 months might have this complication lasting for a long time, suggesting that this group of patients might need extra attention. Additional care to patients with PD early in 3–6 months could mean a more appropriate treatment and improve their quality of life in a long-term after aSAH. All in all, the results showed the improving or the stable trend of PD with time, as other studies both analyzing the acute and chronic phases confirmed [20, 37, 42]. However, some authors [33, 40, 48] reported that an additional hormonal dysfunction may also occur during the follow-up leading to a gradual increase in the prevalence of PD, which was also found in our study. Nevertheless, the mechanism responsible for this difference needs to be further investigated.

A high prevalence of hormonal dysfunction involving the growth hormone and gonadotropin was found in the acute phase, while ACTH deficiency and GHD were more common in the chronic phase. This may be related to the vulnerability of these pituitary endocrine cells to harmful stimuli [49]. In terms of the prevalence of hormonal dysfunction, Can et al. [25] reported a prevalence rate of 19.0% (95% CI, 13.0-26.0%) in GHD after aSAH in the chronic phase, and Dimopoulou et al. [10] reported a long-term prevalence rate of 13% in Gn deficiency and 7% in TSH deficiency, which were similar to our results. Additionally, our results revealed that the prevalence of most hormonal disorders decreased over time, further supporting the decreasing prevalence of PD. However, a slight increase in the prevalence of ACTH deficiency was observed, but taking into consideration tha the 95% confidence intervals overlapped, the difference was not statistically significant and the above conclusion was still valid. The mechanism regulating the changes in these hormonal disorders is not clear, and may be related to the structural hypothalamic-pituitary damage and adaptive mechanisms to acute diseases [21, 50].

The analysis of the WHO Regional Office where the included literature was located revealed that the prevalence of PD after aSAH in the acute phase was the highest in SEARO, which was significantly higher than that in EURO and PAHO. The prevalence of PD in the EURO in the chronic phase was more common than in the other regions. No previous studies on WHO Regional Office in PD after aSAH are available up to now, thus our results on the prevalence of PD in each region could provide a reference for the detection and prevention of PD after aSAH in the corresponding WHO Regional Offices. The result of the prevalence of PD in the EURO was due to the performance of enough studies thanks to the advanced medical level of EURO. The conclusions related to other regions were not enough convincing in view of the small number of studies in those regions ; thus, the number of studies needs to be further increased.

Finally, our results showed that single pituitary hormone dysfunction occurs a little more than three times than that of the multiple, result that was similar to that of previous studies [24], but only in the chronic phase. The prevalence of single hormone dysfunction in the acute phase was slightly higher than that of the multiple, but not statistically significant.

### Limitations

This study has several limitations. The criteria to diagnose PD after aSAH are not unified, meaning that the diagnostic methods were different in diffreent studies. Thus, the large variation in the frequency of hormone deficiencies found in this work from these studies might be due to different methodological approaches for assessing pituitary function. The time to perform the diagnostic test also varied and not all patients were subjected to dynamic testing to assess PD, which may lead to an underestimation of the number of PD patients. The high heterogeneity in our meta-analysis suggests that the pooled prevalence estimates should be interpreted with caution. These estimates may poorly represent the real outcomes without understanding the source of heterogeneity. Potential sources include studies with participants from different countries and the inconsistent diagnostic methods for PD. The heterogeneity is less likely to be explained by WHO region and single/multiple pituitary hormone dysfunction, since it remained high after subgroup analysis. Lastly, only studies available in English were included, which might have influenced the geographic distribution of the included studies.

## Conclusion

In conclusion, our results showed that the prevalence of PD after aSAH decreased over time. The prevalence of the acute phase and chronic phase was 0.50 and 0.30, respectively. Among the hormonal deficiencies, GHD was the most prevalent in the acute phase and ACTH in the chronic phase. Since the inconsistent diagnosis of PD may lead to high heterogeneity among studies, the result in this article should be considered with caution. Thus, multicenter studies with larger sample sizes further clarifying the diagnostic methods should be performed in the future to confirm this result. Some countries have limited research on PD after aSAH, and it is recommended to pay more attention to this disease within the Region of Americas, Eastern Mediterranean Region, Southeast Asia Region, and Western Pacific Region.

## Electronic supplementary material

Below is the link to the electronic supplementary material.

Supplementary Material 1: Additional file 1

Supplementary Material 2: Additional file 2

Supplementary Material 3

Supplementary Material 4

## Data Availability

All information analyzed in this study was available from the corresponding author on reasonable request.
