# Supplementary material for: Prevalence of pituitary dysfunction after aneurysmal subarachnoid hemorrhage: a systematic review and meta-analysis
Source: BMC Neurol. 2023 Apr 20;23:155. doi: 10.1186/s12883-023-03201-x (PMC10116717; doi:10.1186/s12883-023-03201-x)
Supplement: Supplementary file 2 — Supplementary Material 2: Additional file 2 [file 12883_2023_3201_MOESM2_ESM.docx]

**ADDITIONAL FILE 2**

Sensitivity analysis of the pooled prevalence of PD in the chronic phases after aSAH.
